# The Potential Distribution of *Termitomyces* spp. in China Based on MaxEnt Model Analysis

**DOI:** 10.1002/ece3.71477

**Published:** 2025-06-01

**Authors:** Dan Yong, Danping Xu, Xinqi Deng, Zhipeng He, Biyu Liu, Xuezhen Yang, Zhihang Zhuo

**Affiliations:** ^1^ College of Life Science China West Normal University Nanchong China; ^2^ Sichuan Institute of Edible Fungi Sichuan Academy of Agricultural Sciences Chengdu China

**Keywords:** China, climate change, habitat suitability prediction, MaxEnt, *Termitomyces* spp.

## Abstract

*Termitomyces* spp., as a precious edible and medicinal fungus, has long been a focal point of attention due to its unique distribution characteristics and cultivation potential, which have sparked extensive research and discussion. To effectively predict the suitable growth areas and cultivation environments for *Termitomyces* spp., this study combines the MaxEnt model and ArcGIS software to systematically predict its potential distribution patterns under climate change. By analyzing the relationship between the geographic distribution of *Termitomyces* spp. and bioclimatic factors, the study reveals that temperature and precipitation are key climate factors influencing its distribution, with warm and humid climate conditions promoting its growth and reproduction. The model results show that under current climate conditions, the total suitable habitat area for *Termitomyces* spp. reaches 152.95 × 10^4^ km^2^, primarily distributed in southwestern, southern, central, and eastern China. With future climate scenarios, the potential suitable habitats for *Termitomyces* spp. show a trend of gradual expansion, even covering the northwestern regions. The overall suitable habitat area significantly increases, with only a slight decrease in the area of moderately and low‐suitability zones. Additionally, the study also finds that the centroid of the suitable habitats tends to migrate in the northwest direction. In conclusion, the findings of this study not only provide scientific evidence for exploring the impact of climate change on the distribution of *Termitomyces* spp., but also offer important data support for optimizing its cultivation techniques, promoting ecological conservation efforts, and formulating regional economic development strategies.

## Graphical abstract

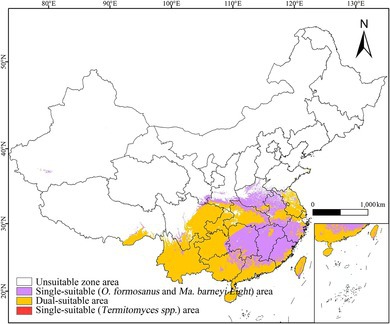

## 1 Introduction

Mushrooms refer to large fungi that can form tough fungal tissues or large, fleshy structures, and are widely distributed across the globe (Hawksworth 2001; Zhang et al. 2021). They are an indispensable part of nature. Currently, among the 16,000 known edible mushrooms, about 7000 species are edible for humans, of which approximately 3000 are widely distributed in the market, and around 700 species are highly valued for their unique medicinal properties (Li et al. 2021; Hawksworth 2012). The *Termitomyces* spp. species, studied in this article, is one of the outstanding examples. *Termitomyces* belongs to the Basidiomycota division (Yang et al. 2019; Yue et al. 2010). It is not only loved for its delicious taste and rich nutritional content (Paloi et al. 2023), but also for its significant pharmacological effects due to its bioactive compounds, such as antioxidant, immune modulation, ulcer healing, and analgesic properties (Hsieh and Ju 2018; Liu et al. 2021).

*Termitomyces* has relatively high requirements for its growth environment. It thrives at temperatures between 24°C and 30°C, with a light intensity of 500–1000 Lux, and a soil pH of 4–4.5. Its fruiting bodies can only grow on termite nests, making artificial cultivation challenging. It requires a natural environment where it can coexist with termites. This unique growth pattern has led to a close ecological relationship between *Termitomyces* and termites (Binbin et al. 2024).

The Macrotermitinae, which belongs to the Termitidae (Darlington et al. 2008), consists of termites that can cultivate fungi within their nests, and thus these termites are also known as fungus‐growing termites (Chen et al. 2021). A sophisticated mutualistic symbiotic relationship exists between fungus‐growing termites and *Termitomyces*: on the one hand, the termite nests maintain relatively stable humidity and temperature, providing a suitable environment for *Termitomyces* to grow; on the other hand, *Termitomyces* helps the fungus‐growing termites degrade their primary food sources—plant matter such as wood, hay, and leaf litter—providing necessary nutrition (Rouland‐Lefèvre et al. 2006). This complementary relationship makes the two species interdependent within the ecosystem, essential for each other's survival and reproduction.

However, with the increasing intensity of human activities, particularly the overharvesting of *Termitomyces*, their survival environment is facing unprecedented challenges. In the pursuit of this precious resource, people often destroy termite nests, a practice that not only directly threatens the habitats of the fungus‐cultivating termites but also severely disrupts their ecological balance, leading to a significant decline in termite populations (Sitotaw et al. 2020). At the same time, the ongoing impacts of climate change have further exacerbated the survival pressure on *Termitomyces*, subjecting their natural distribution to dual threats and making their survival environment more fragile and unpredictable. In this context, by studying the potential distribution areas of *Termitomyces* under future climate change conditions, we can provide important theoretical foundations and practical guidance for their sustainable development and the realization of their multifaceted benefits, thereby laying a scientific basis for the protection of this unique species and its ecosystem.

As *Termitomyces* is mainly found in the wild in China (Ye et al. 2019), its natural distribution is influenced by multiple factors, such as ecological environment and climate change (Koné et al. 2018). Therefore, predicting its potential distribution area is particularly important. Species Distribution Models (SDMs) are essential tools in ecological research. They integrate species distribution data with environmental variables to accurately predict the potential distribution areas of species (Tan et al. 2023; Booth 2018). In the field of ecology, the application of SDMs is widespread and profound, playing a crucial role in species conservation and the formulation of ecological restoration strategies. By predicting species distribution, SDMs not only help optimize conservation measures but also provide scientific evidence for the design of ecological restoration plans (Rathore and Sharma 2023).

Compared to traditional SDMs, the MaxEnt model stands out when dealing with scarce data (Phillips et al. 2006). It can predict the potential impact of future climate change on species distribution and identify the main climate factors influencing species distribution, such as temperature and precipitation (Zhang et al. 2018; Li et al. 2020), which directly affect species survival and expansion. The basic principle of MaxEnt is to use known species distribution data and relevant environmental variables to calculate the ecological requirements of the species through an algorithm, then map the results to different spaces and times to predict the species' actual and potential distribution (Gengping et al. 2013). Moreover, the accuracy and stability of MaxEnt make it one of the most frequently used SDMs, widely applied in invasion ecology, conservation biology, and studying the effects of global climate change on species distribution (Gengping et al. 2013; Peterson et al. 2011). In conclusion, using MaxEnt to predict the potential distribution of *Termitomyces* in China is highly applicable. In fact, many studies have already used MaxEnt to successfully predict the distribution of important medicinal fungi in China, such as the potential distribution of *Phellinus igniarius* (Yuan et al. 2015), the potential suitable planting areas for *Ganoderma lucidum*, and the suitable planting areas for Poria cocos in Jinzhai County, Dabie Mountains.

Currently, research on *Termitomyces* in China mainly focuses on its symbiotic relationship with fungus‐growing termites, species identification, and the optimization of artificial cultivation techniques (Wood and Thomas 1989; Wei et al. 2009; Aryal 2020). These studies provide a rich theoretical foundation for understanding the ecological characteristics of *Termitomyces* and its artificial cultivation. However, studies on the distribution characteristics and influencing factors of *Termitomyces*' potential suitable habitats in China remain limited. Although the interactions between *Termitomyces* and fungus‐growing termites show significant ecological effects in natural environments (Thomas 1981), this symbiotic relationship depends on specific ecological conditions and climatic environments (Chen et al. 2021; Vesala et al. 2019). Existing research has not fully explored the variation patterns of its potential habitats and the driving factors behind them (Pradhan et al. 2013). Therefore, this study aims to fill this gap by using the geographical distribution data of *Termitomyces* and predicting its potential suitable distribution areas in China through the MaxEnt model. Through this research, we can not only identify areas where *Termitomyces* may expand in the future but also reveal the potential impact of climate change on its habitat suitability. Further analysis will help assess the far‐reaching impact of climate change on *Termitomyces* distribution, providing important data support for optimizing cultivation techniques, promoting ecological conservation, and developing regional economic strategies.

## 2 Materials and Methods

### 2.1 Data Sources and Distribution Point Processing of *Termitomyces*

To establish a reliable morphological classification system for the species and distribution of *Termitomyces* in China, Wei Tiezheng and colleagues reexamined, compared, and identified *Termitomyces* specimens housed in major domestic herbarium collections, ultimately confirming 13 species distributed within China (Tiezheng 2004). This systematic classification laid a solid foundation for subsequent research. To ensure the comprehensiveness and accuracy of the data, we employed multiple methods for strict data collection and screening: (1) First, we systematically collected research materials related to *Termitomyces* by reviewing domestic and international literature databases and extracted valid distribution information; (2) Second, we conducted online data searches using Google Scholar (https://scholar.dosf.top/) and the Global Biodiversity Information Facility database (https://www.gbif.org) to ensure that the latest research results and data were included; (3) For sample points lacking latitude and longitude information but with detailed geographical location records (such as precise townships or more accurate geographic markers), we used Google Maps to query the relevant regional coordinates and obtained their accurate latitude and longitude data.

**FIGURE 1:**
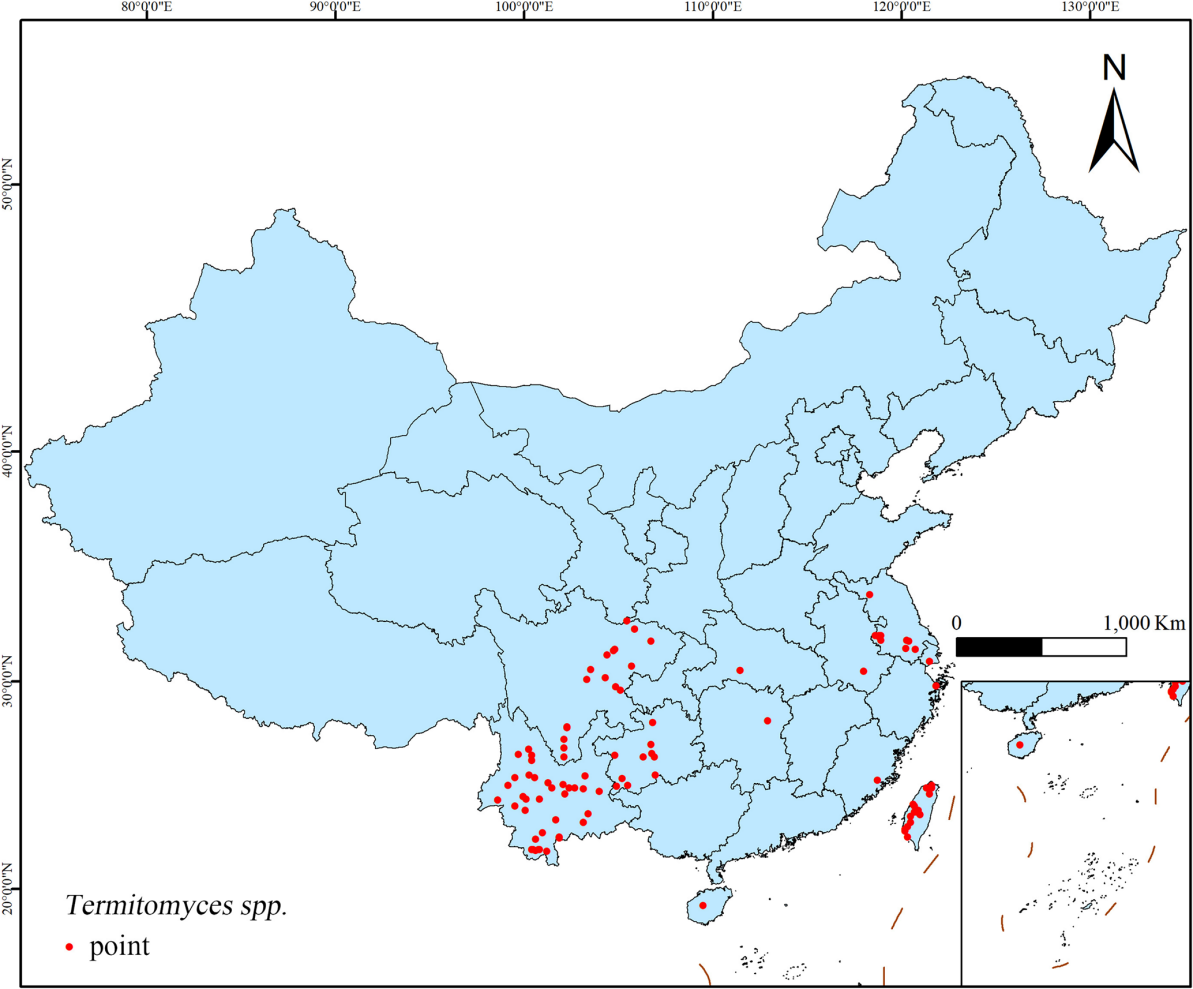
The distribution points of *Termitomyces* in China.

This study collected a total of 114 distribution points for *Termitomyces*, including *Termitomyces* R. Heim (62 points), *Termitomyces microcarpus* (Berk. & Broome) R. Heim (18 points), *Termitomyces eurrhizus* (Berk.) R. Heim (17 points), *Termitomyces aurantiacus* (R. Heim) R. Heim (two points), *Termitomyces medius* R. Heim & Grassé (one point), *Termitomyces striatus* (Beeli) R. Heim (two points), *Termitomyces clypeatus* R. Heim (three points), *Termitomyces albuminosus* (Beck.) Heim (three points), *Termitomyces intermedius* Har. Takah. & Taneyama (four points), and *Termitomyces bulborhizus* T. Z. Wei, Y. J. Yao, Bo Wang & Pegler (two points). To effectively reduce sampling bias caused by clustering effects and eliminate redundant data to avoid model overfitting, we utilized the ENMTools software to perform precise spatial filtering on the species distribution data. During this process, we retained only the most representative distribution point within each 5 km × 5 km grid cell, significantly enhancing the spatial independence and accuracy of the data. The processed data were exported in .csv format to support subsequent MaxEnt model development and analysis. Through this series of rigorous data processing steps, we ultimately constructed a high‐quality dataset containing 96 distribution records, which are more evenly distributed spatially and highly representative. Based on the processed data, we further used ArcGIS 10.8 software to create a detailed geographic distribution map of *Termitomyces* (Figure 1).

### 2.2 Climate Data Selection and Preprocessing

This study constructs SDMs using two environmental factors: Topography and climate, in order to more accurately simulate the distribution patterns of the target species. The current 19 bioclimatic factor data, future climate data (2050s, 2070s), and one topographic factor (elevation) are all sourced from the WorldClim database (https://www.Worldclim.org/; Elith and Leathwick 2009). For future climate scenarios, data from the Coupled Model Intercomparison Project Phase 6 (Meinshausen et al. 2017) were used, specifically the climate scenario data from the Beijing‐based National Climate Center's climate system model (BCC‐CSM2‐MR) under the SSP1‐2.6, SSP2‐4.5, and SSP5‐8.5 scenarios.

In data processing, we used the masking and clipping tool in ArcGIS 10.8 to spatially clip the aforementioned variables, and then converted the clipped raster data into ASCII format to meet the input requirements of the MaxEnt model (Oymatov et al. 2023). Subsequently, we used ENMTools software to calculate the Pearson correlation coefficient (*r*) for the relevant environmental factors, in order to detect multicollinearity. Environmental factors with a correlation coefficient > 0.8 (|*r*| > 0.8) were removed, and the remaining environmental factors with relatively low correlation were selected and imported into the MaxEnt software for modeling. After four rounds of screening, nine key environmental variables (bio2, bio3, bio4, bio8, bio9, bio14, bio15, bio18, and elev) were finalized as the input factors for the model construction (Table 1).

**TABLE 1:** Description of environmental variables.

Symbol	Environmental variable	Unit
Mean diurnal range (mean of monthly) (max temp–min temp)	bio2	°C
Isothermality (bio2/bio7) (*100)	bio3	°C
Temperature seasonality (standard deviation*100)	bio4	°C
Mean temperature of Wettest Quarter	bio8	°C
Mean temperature of Driest Quarter	bio9	°C
Precipitation of Driest Month	bio14	mm
Precipitation seasonality (coefficient of variation)	bio15	%
Precipitation of Warmest Quarter	bio18	mm
Altitude	elev	m

### 2.3 Construction and Optimization of the MaxEnt Model

The cross‐validation method was used to extract the test set, dividing the distribution records obtained above into 10 subsets, with one subset used as the test set and the remaining nine subsets used as the training set, repeating the process 10 times. The potential habitats were classified into four levels using the equal interval method: Unsuitable habitat (suitability value 0–0.30), which provides minimal support for the growth of *Termitomyces*; low suitability habitat (0.30–0.50), which may support the survival of *Termitomyces* under certain conditions; moderately suitable habitat (0.50–0.70), which is suitable for the reproduction and growth of *Termitomyces*; and highly suitable habitat (> 0.70), which is highly suitable for the reproduction and growth of *Termitomyces*. At the same time, the model's accuracy was evaluated using the receiver operating characteristic (ROC) curve. The area under the curve (AUC) value ranges from 0 to 1, with higher AUC values indicating more accurate model predictions.

In recent years, numerous studies have indicated that the default settings of the MaxEnt model, primarily based on empirical adjustments, may lead to suboptimal model performance (Radosavljevic and Anderson 2014). To enhance the predictive capability of the model, this study optimized the selection of original predictor variables (“feature classes” or FC) by increasing the combination of feature classes to improve the model's flexibility and fitting ability. Specifically, a blocking method was employed to divide 98 *Termitomyces* distribution records into four equal parts, with three parts used for training and one part for testing. Additionally, 13 regularization multiplier (RM) parameters and six feature class (FC) parameter combinations (including L, LQ, H, LQH, LQHP, and LQHPT, where L represents linear, Q represents quadratic, H represents hinge, P represents product, and T represents threshold) were set. The ENMeval package in R software was used to test the aforementioned 78 parameter combinations, and the Akaike Information Criterion corrected (AICc) was applied to evaluate the model's fit and complexity. The parameter combination with the smallest delta.AICc value was ultimately selected as the optimal model. After optimization, the final parameter combination determined was feature class LQ (linear + quadratic) with a RM (rm = 1), using 25% of the data as the test set and repeating the runs 10 times to ensure the model's stability and reliability.

### 2.4 Changes in the Centroid of *Termitomyces* Under Future Climate Scenarios

This study focuses on the suitable habitats of *Termitomyces*, examining the spatial changes of its potential suitable areas in China by analyzing the shifts in the centroid of suitable habitats under current and future climate scenarios. We used ArcGIS 10.8 software to convert the suitability distribution maps from the MaxEnt model into binary format to obtain the centroid coordinates. By connecting the centroids of suitable habitats under different climate scenarios, we further reveal the spatial variation trend and evolutionary direction of the suitable habitats for *Termitomyces*.

## 3 Results

### 3.1 Optimization Process and Accuracy Evaluation of the MaxEnt Model

**FIGURE 2:**
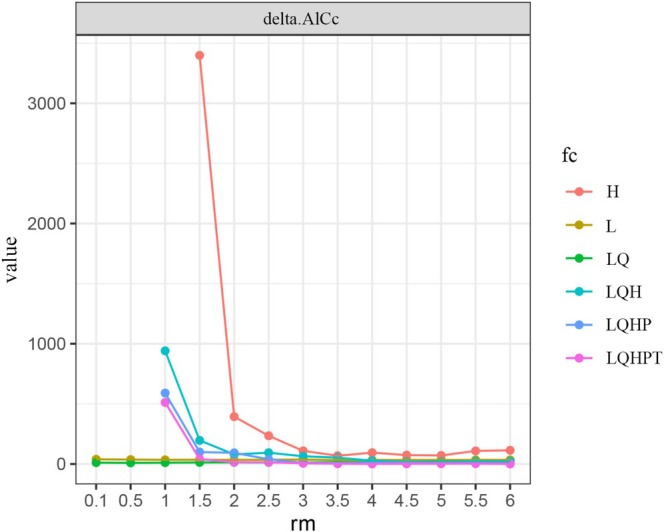
MaxEnt model parameter combination.

**FIGURE 3:**
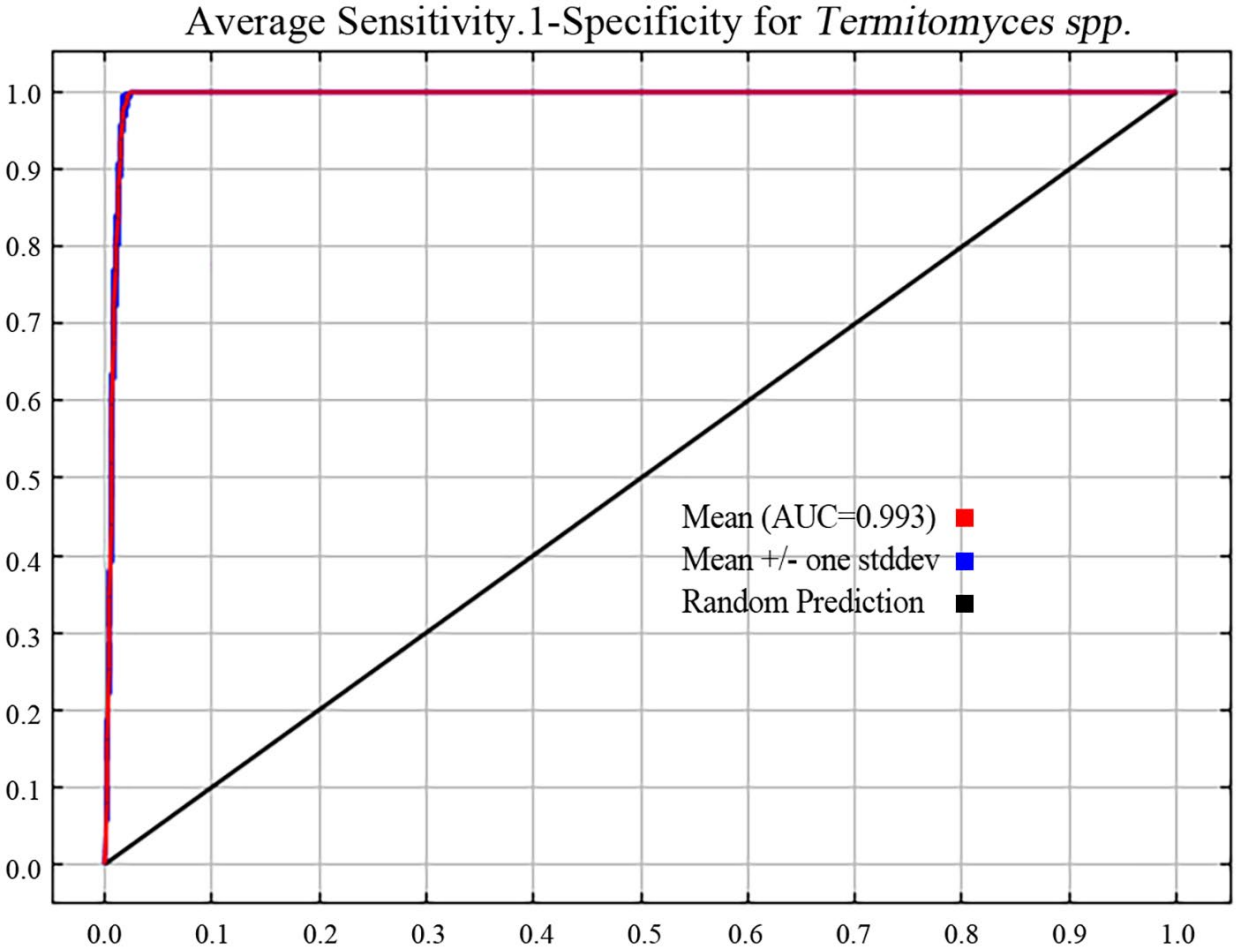
The receiver operating characteristic curve predicting the current potential distribution of *Termitomyces* based on the MaxEnt model.

By using the ENMeval package to optimize the MaxEnt model parameters, it can be seen from Figure 2 (Delta.AICc results of the MaxEnt model for *Termitomyces* under different parameter combinations) that when RM = 0.1 and FC = LQ, delta.AICc = 0. According to the Akaike Information Criterion, this parameter combination results in the lowest model complexity and minimal overfitting. Therefore, RM = 0.1 and FC = LQ were selected as the optimal model parameters. Using the optimized parameters, the model was reconstructed to simulate the suitable habitat for *Termitomyces*. The AUC value of the training simulation with these parameters was 0.993, significantly higher than 0.9 (Figure 3), far exceeding the AUC value of the random prediction model. This indicates that the model performs excellently in terms of prediction accuracy and can accurately reflect the potential distribution of *Termitomyces* under current and future climate scenarios.

### 3.2 Important Climatic Factors Affecting the Distribution of *Termitomyces*

**FIGURE 4:**
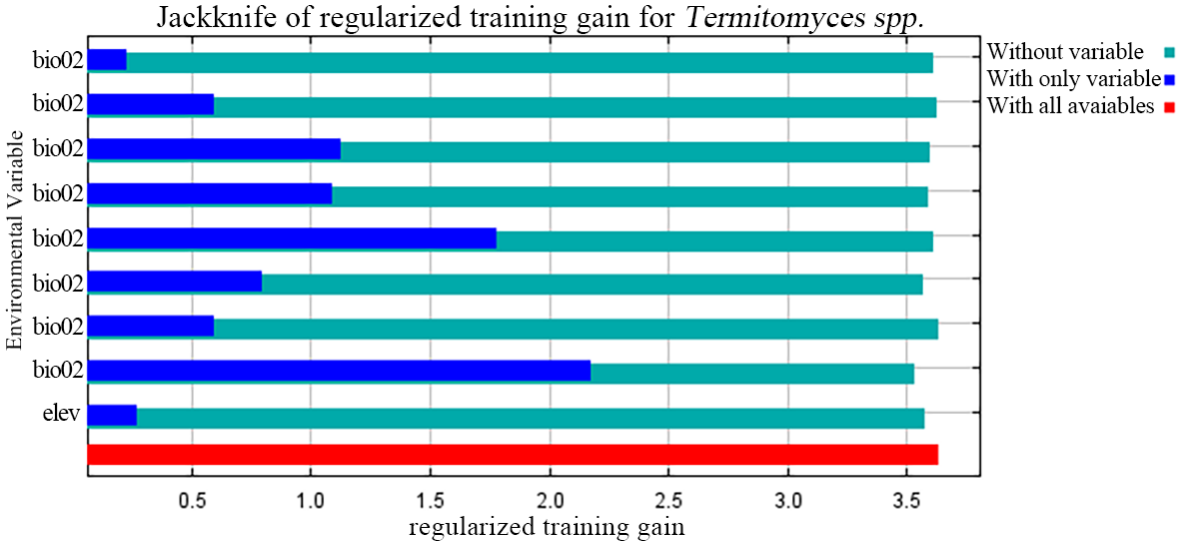
Jackknife test for climatic factors in the current potential distribution model of *Termitomyces*.

Based on nine environmental variables, we ran the MaxEnt model and evaluated the percentage contribution, permutation importance, and confidence intervals of each climate factor (see Table 2). The analysis results show that the top four climate factors with the highest percentage contribution are precipitation of the warmest quarter (Bio18), temperature seasonality (Bio04), precipitation seasonality (Bio15), and mean temperature of the driest quarter (Bio09), with confidence intervals of [15.3, 18.1], [6.1, 7.7], [0.0, 0.2], and [20.5, 23.7], respectively. The top four climate factors with the highest permutation importance are mean temperature of the wettest quarter (Bio08), mean temperature of the driest quarter (Bio09), precipitation of the warmest quarter (Bio18), and temperature seasonality (Bio04), with confidence intervals of [45.2, 50.2], [20.5, 23.7], [15.3, 18.1], and [6.1, 7.7], respectively. Further analysis using the Jackknife test on the model scores of different climate factors (Figure 4) revealed that the top four climate factors are precipitation of the warmest quarter (Bio18), mean temperature of the driest quarter (Bio09), temperature seasonality (Bio04), and mean temperature of the wettest quarter (Bio08). In conclusion, Bio18, Bio09, Bio04, and Bio08 are key climate factors influencing the distribution of *Termitomyces*, and their confidence intervals further support the importance of these factors.

**TABLE 2:** The contribution rate and permutation importance of each climatic factor to the distribution of *Termitomyces*.

Variables	Percent contribution	Permutation importance/%	Confidence interval (95%)
**Bio8**	**0.6**	**47.7**	**[45.2, 50.2]**
**Bio9**	**4.4**	**22.1**	**[20.5, 23.7]**
**Bio18**	**63.7**	**16.7**	**[15.3, 18.1]**
**Bio4**	**17.3**	**6.9**	**[6.1, 7.7]**
Elev	2.3	2.6	[2.1, 3.1]
Bio2	2.3	2.1	[1.8, 2.4]
Bio14	4.4	1.5	[1.2, 1.8]
Bio15	4.7	0.1	[0.0, 0.2]
Bio3	0.2	0.3	[0.1, 0.5]

*Note:* Bold values (Bio8, Bio9, Bio18, Bio4) indicate the key climatic factors influencing the distribution of Termitomyces fungi. Their statistical significance is further supported by 95% confidence intervals.

### 3.3 Adaptive Response to Climatic Factors

**FIGURE 5:**
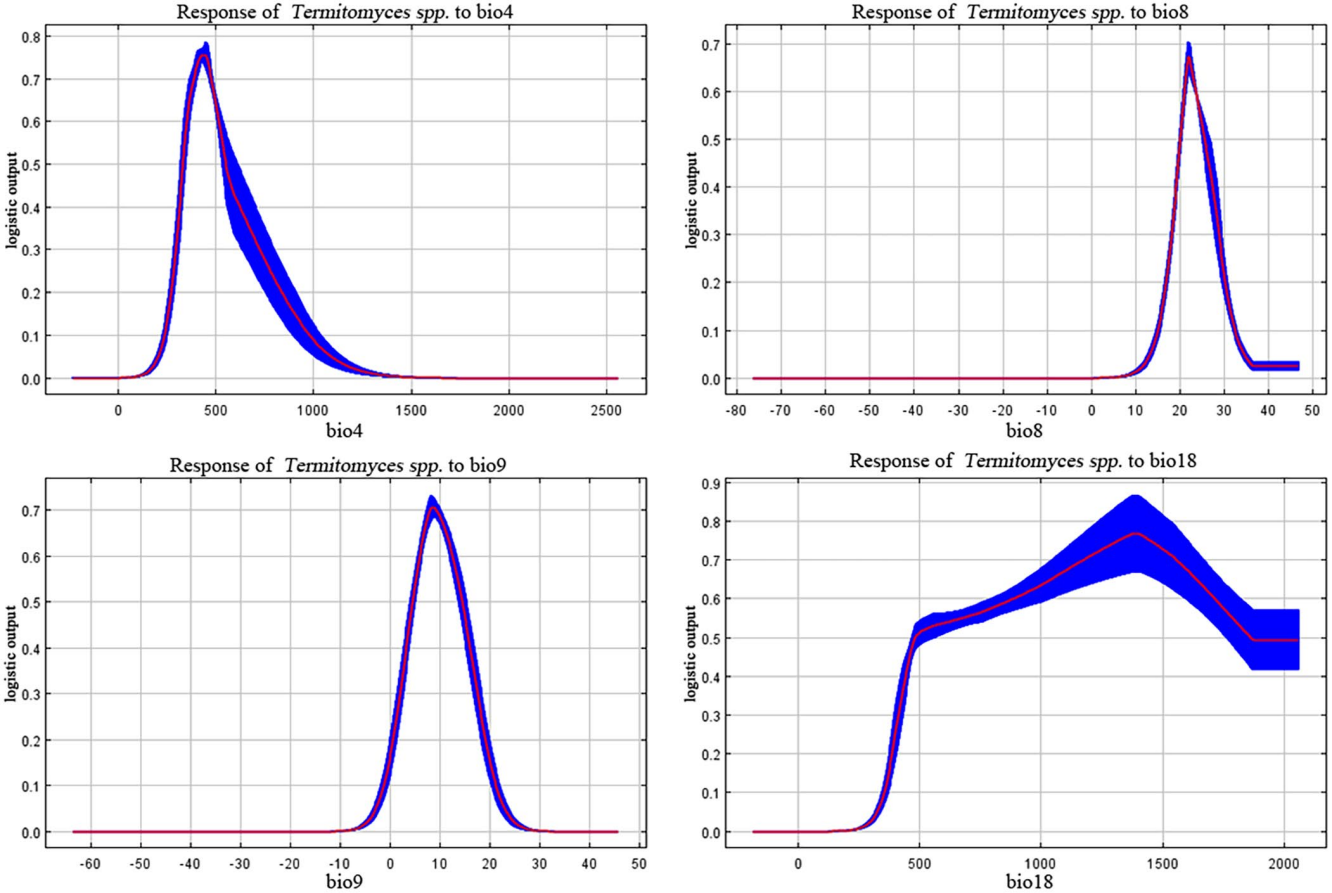
Probability response curves of the main climatic factors.

Based on the results from the MaxEnt model, we plotted the response curves of *Termitomyces* suitability to the four climatic factors mentioned above (Figure 5). The probability of presence of *Termitomyces* in response to each climatic factor shows a trend where the probability initially increases rapidly and then decreases slowly as the value of the climatic factor increases. In this study, the range for selecting climatic factors with a probability of presence > 0.5 represents the climatic characteristics of the distribution area of *Termitomyces*. The climatic characteristics of the distribution area of *Termitomyces* are as follows: The temperature seasonality (Bio04) ranges from 328.5°C to 543.1°C, with the optimal value at 435.0°C; the mean temperature of the wettest quarter (Bio08) ranges from 20.1°C to 25.5°C, with the optimal value at 22.2°C; the mean temperature of the driest quarter (Bio09) ranges from 4.7°C to 15.0°C, with the optimal value at 8.4°C; and the precipitation of the warmest quarter(Bio18) ranges from 483.7 to 1846.5 mm, with the optimal value at 1389.28 mm.

### 3.4 Potential Suitable Habitat Distribution of *Termitomyces* in China Under Current Climate Scenario

**FIGURE 6:**
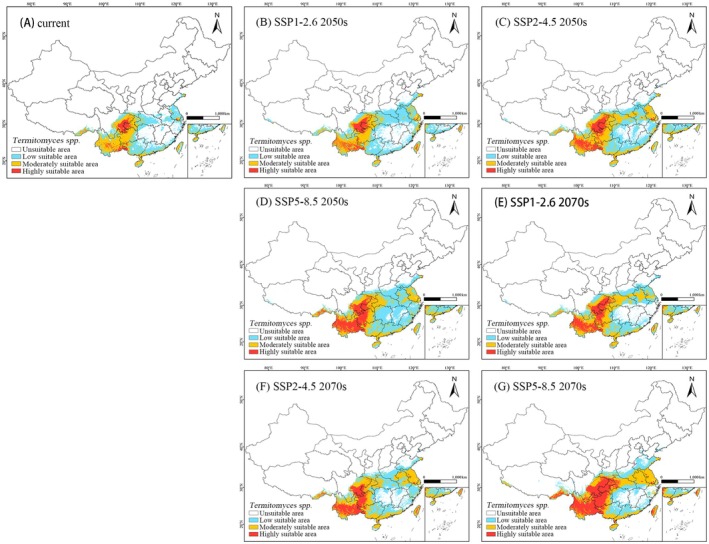
Potential and future distribution ranges of *Termitomyces* in China under current and projected climate scenarios predicted by MaxEnt. White, unsuitable habitat area; light blue, low habitat suitability area; yellow, moderate habitat suitability area; red, highly habitat suitability area.

Based on the prediction results of the MaxEnt model, under the current climate scenario, the total suitable habitat of *Termitomyces* is mainly concentrated in the southwestern, southern, central, and eastern regions of China. The highly suitable habitats are primarily distributed in the central and eastern parts of Yunnan Province, the western part of Guangxi Province, the eastern part of Sichuan Province, the western part of Chongqing City, the northern part of Guizhou Province, the southern part of Jiangsu Province, the central part of Taiwan, the southeastern part of Tibet Autonomous Region, and the western part of Hainan. In addition to these highly suitable areas, parts of Yunnan Province, Guizhou Province, Chongqing City, eastern Sichuan Province, southeastern Tibet Autonomous Region, western Guangxi Zhuang Autonomous Region, southern Guangdong Province, Hainan Province, and most of Taiwan are considered moderately suitable habitats. The low‐suitability areas extend from the south of Hainan Province to the southern parts of Shaanxi and Henan Provinces, westward to the southeastern part of Tibet Autonomous Region, eastward to Jiangsu Province, Zhejiang Province, and Taiwan, with a small distribution in the eastern part of Shandong Province (Figure 6A).

Under the current climate scenario, the total suitable habitat area for *Termitomyces* is 152.95 × 10^4^ km^2^, accounting for 15.93% of China's land area (Table 3). Among this, the highly suitable habitat area is 15.05 × 10^4^ km^2^, making up 9.83% of the total suitable habitat area; the moderately suitable habitat area is 65.13 × 10^4^ km^2^, accounting for 42.58% of the total suitable habitat area; and the low‐suitability habitat area is 72.77 × 10^4^ km^2^, which represents 47.57% of the total suitable habitat area.

**TABLE 3:** Prediction of the suitable habitat area for *Termitomyces* in China under current and future climate conditions (km^2^).

Classification level^a^		Suitable habitat (km^2^) (0.3–1)	Low habitat suitability (km^2^) (0.3–0.5)	Moderate habitat suitability (km^2^) (0.5–0.7)	High habitat suitability (km^2^) (0.7–1)	Confidence interval (95%)
Current climate		~152.95 × 10^4^	~72.77 × 10^4^	~65.13 × 10^4^	~15.05 × 10^4^	[149.50, 156.40] × 10^4^
Future climate conditions	SSP1‐2.6‐2050s	~186.06 × 10^4^ (21.65%)^b^	~101.22 × 10^4^ (39.09%)	~64.37 × 10^4^ (−1.16%)	~20.47 × 10^4^ (36.01%)	[182.00, 190.12] × 10^4^
	SSP2‐4.5‐2050s	~212.08 × 10^4^ (38.66%)	~92.91 × 10^4^ (27.68%)	~87.66 × 10^4^ (34.59%)	~31.51 × 10^4^ (109.37%)	[207.00, 217.16] × 10^4^
	SSP5‐8.5‐2050s	~269.87 × 10^4^ (76.44%)	~113.10 × 10^4^ (73.65%)	~113.10 × 10^4^ (73.65%)	~43.66 × 10^4^ (190.09%)	[264.00,275.74] × 10^4^
	SSP1‐2.6‐2070s	~188.49 × 10^4^ (23.24%)	~74.31 × 10^4^ (2.11%)	~77.44 × 10^4^ (18.09%)	~36.74 × 10^4^ (144.12%)	[184.00, 193.00] × 10^4^
	SSP2‐4.5‐2070s	~204.75 × 10^4^ (33.87%)	~84.02 × 10^4^ (15.46%)	~83.01 × 10^4^ (27.45%)	~37.72 × 10^4^ (150.63%)	[199.00, 210.50] × 10^4^
	SSP5‐8.5‐2070s	~223.48 × 10^4^ (46.11%)	~71.82 × 10^4^ (−1.3%)	~81.20 × 10^4^ (24.67%)	~70.47 × 10^4^ (368.24%)	[219.00, 228.00] × 10^4^

^a^ Suitable and unsuitable habitat of *Termitomyce*s of all suitable distribution areas.

^b^ The brackets show the proportion of the area of the suitable area under future climate scenarios relative to the area of the suitable area under the current climate scenario.

### 3.5 Potential Suitable Habitat Distribution of *Termitomyces* in China Under Future Climate Scenarios

Based on the MaxEnt model, this study predicts the potential suitable habitat distribution of *Termitomyces* under future climate scenarios (Table 3, Figure 6B–G). The results show that the potential suitable habitats of *Termitomyces* gradually expand, extending into the northwest region. Specifically, by the 2050s, the area of suitable habitats for *Termitomyces* significantly increases under all climate scenarios. Except for a 1.16% decrease in the area of moderate suitability under the SSP1‐2.6 scenario, both low and high‐suitability areas show significant growth. Particularly under the SSP5‐8.5 scenario, the area of high‐suitability increases by as much as 190.09%. The expansion of high‐suitability areas is mainly concentrated in Yunnan, Guizhou, Sichuan, and Taiwan provinces, whereas the increase in moderate suitability areas is mainly observed in Hubei, Henan, Anhui, and Jiangsu provinces. The expansion of low suitability areas is primarily concentrated in Henan, Anhui, Hunan, Jiangxi, and Fujian provinces.

By the 2070s, the area of suitable habitats for *Termitomyces* continues to significantly increase across all periods. Except for a 1.3% decrease in the low‐suitability area under the SSP5‐8.5 scenario compared to the present, the area of all other suitability levels significantly expands, especially under the SSP5‐8.5 scenario, where the high‐suitability area increases by as much as 368.24%. At this point, the growth of high‐suitability areas is primarily concentrated in Yunnan, Sichuan, Guizhou, and Chongqing; the increase in moderate suitability areas is mainly observed in Hubei, Anhui, Guangdong, and Jiangsu; and the expansion of low‐suitability areas is concentrated in Henan, Anhui, Zhejiang, and Shandong provinces.

### 3.6 Centroid Shifts of Potential Suitable Habitats for *Termitomyces* Under Climate Change Scenarios

**FIGURE 7:**
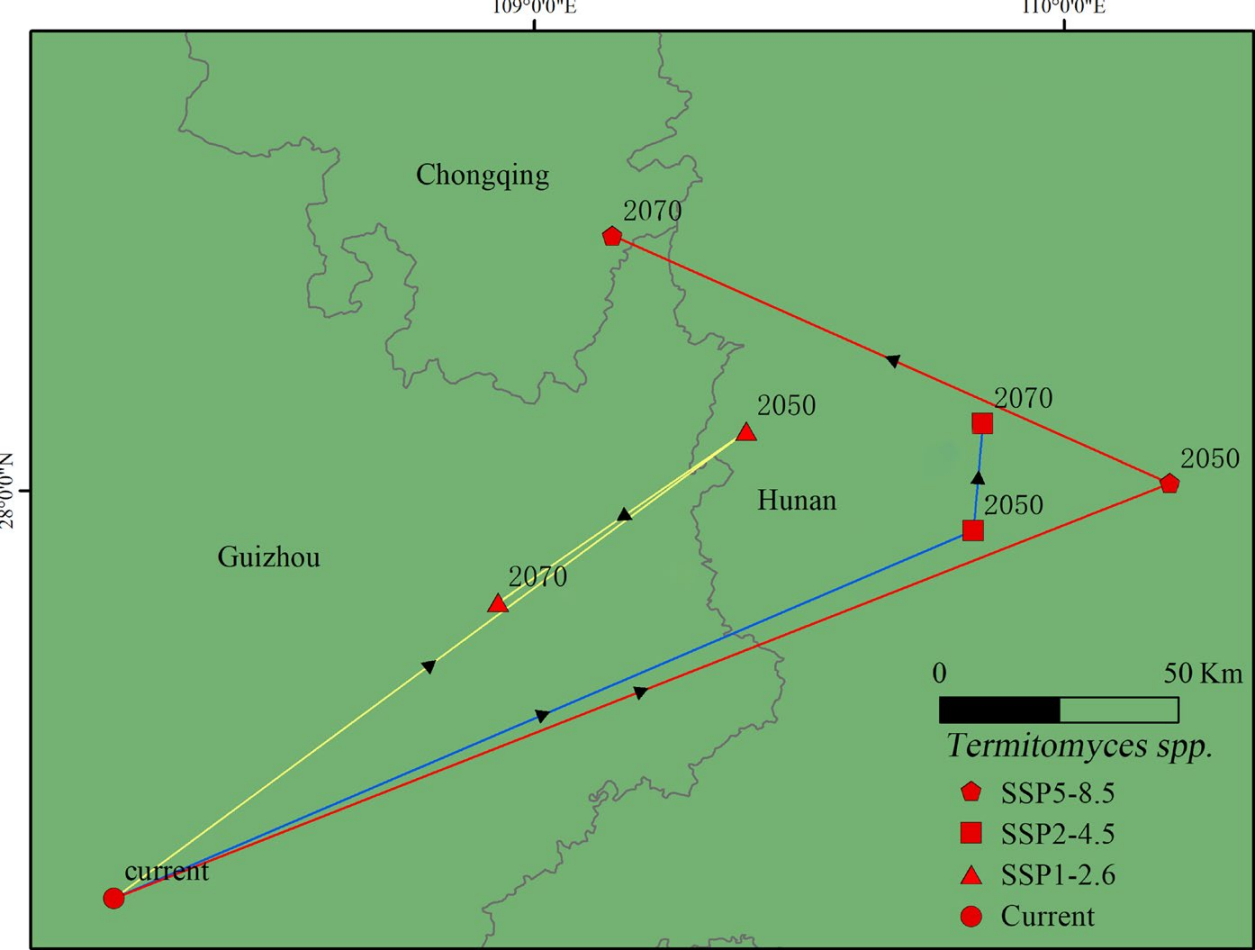
Changes in the centroid of the potential suitability areas for *Termitomyces* in China.

The changes in the centroid of the total suitable habitat for *Termitomyces* under current and future climate conditions are shown in Figure 7. Under current climate conditions, the centroid of *Termitomyces* is located in Guizhou Province (108.21° E, 27.23° N). Under the SSP1‐2.6 scenario, the centroid shifts from western Hunan Province (109.40° E, 28.11° N) in the 2050s to eastern Guizhou Province (108.93° E, 27.79° N) in the 2070s. Under the SSP2‐4.5 scenario, the centroid moves from Hunan Province (109.83° E, 27.92° N) in the 2050s to northern Hunan Province (109.85° E, 28.13° N) in the 2070s. Under the SSP5‐8.5 scenario, the centroid shifts from Hunan Province (110.20° E, 28.01° N) in the 2050s to Chongqing City (109.15° E, 28.48° N) in the 2070s. Overall, the suitable habitat for *Termitomyces* shows a trend of shifting northwestward.

## 4 Discussion

With the continuous development of geographic information systems (GIS) and the application of new statistical techniques, SDM have evolved into various niche models, such as the genetic algorithm for rule‐set prediction, generalized linear models, MaxEnt, generalized additive models, and others (Townsend Peterson et al. 2007; Domene et al. 2012; Xu et al. 2019; Zuur et al. 2009). Among these models, MaxEnt has become one of the most widely used due to its excellent accuracy and stability in recent years (Peterson et al. 2011). However, in the practical application of the MaxEnt model, researchers often use default parameters for model construction. These default parameters stem from early tests conducted by the developers based on data from six different geographical regions and 266 species, aiming to simulate species distribution (Phillips and Dudík 2008). However, subsequent studies have shown that the complex machine learning algorithms used in the MaxEnt model are sensitive to sampling biases, which can lead to overfitting and reduce the model's transferability. Zhu Gengping et al. noted that, compared to default parameters, adjusting the MaxEnt model using the ENMeval package in R software, by modifying the frequency and feature combination parameters, can produce a better predictive model. This model yields smoother response curves and stronger transferability, more accurately reflecting the species' responses to environmental factors and precisely simulating their potential distribution (Gengping and Huijian 2016).

**FIGURE 8:**
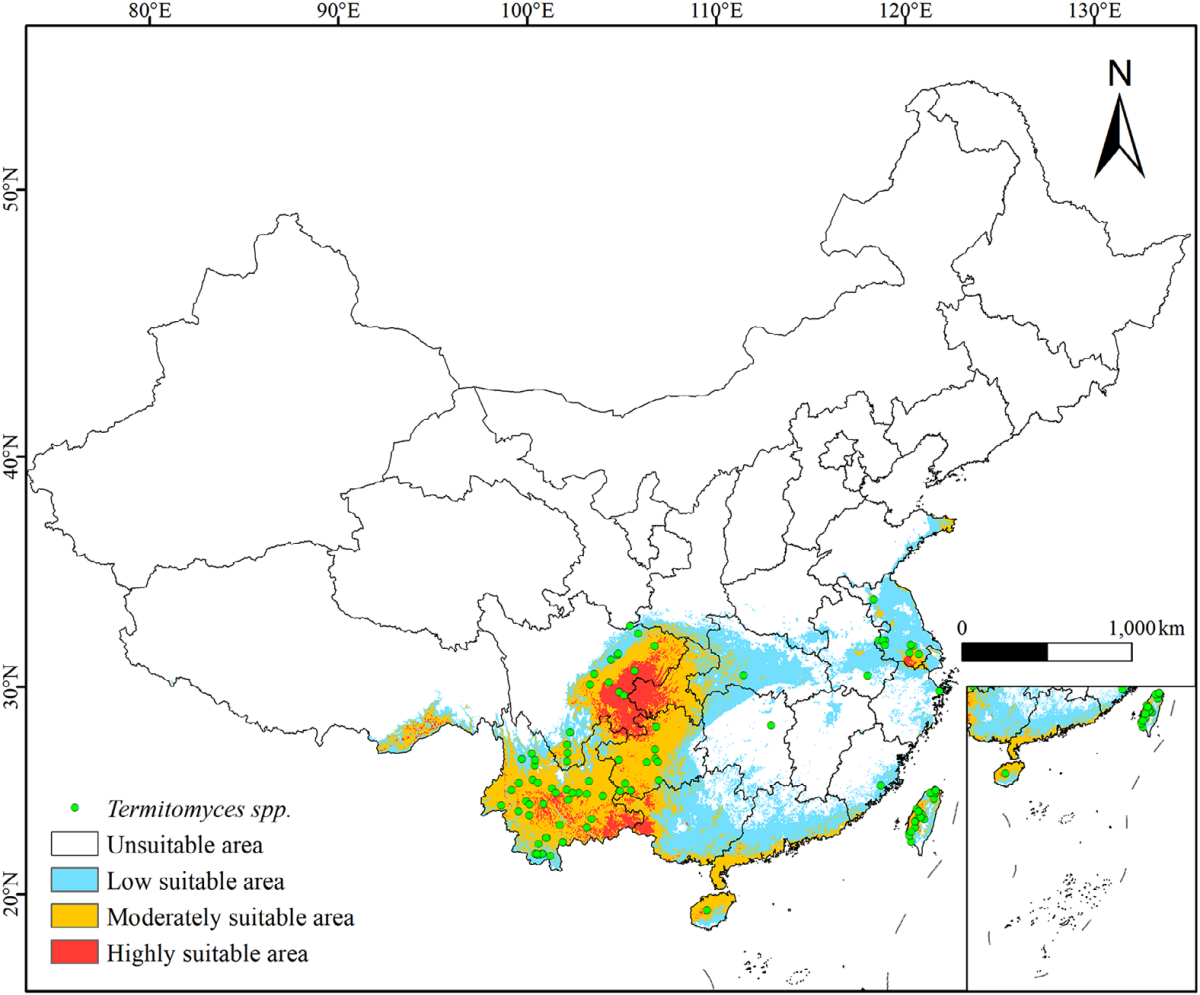
Comparative analysis of current predicted suitable habitats and historical observed distributions. White, unsuitable habitat area; light blue, low habitat suitability area; yellow, moderate habitat suitability area; red, highly habitat suitability area.

In this study, we implemented a series of rigorous measures to ensure the accuracy and reliability of the model predictions. Firstly, to reduce the potential impact of sampling bias on the model predictions, we rigorously filtered the distribution points of *Termitomyces* using the ENMTools software. Secondly, to address potential multicollinearity issues among climate variables, we selected key bioclimatic variables with minimal impact on the model by combining correlation analysis and the Jackknife test. Additionally, to prevent overfitting in the MaxEnt model, we meticulously optimized the model's RMs and feature parameters. The validation results of the model predictions showed a high consistency between the test omission rate and the theoretical omission rate, indicating low spatial autocorrelation between the selected species distribution points and climate variables, and demonstrating the model's high precision and credibility. To further validate the accuracy of the habitat expansion predictions, we comprehensively evaluated the model's predictions by integrating historical distribution records of *Termitomyces* and past climate conditions, and compared the currently predicted suitable habitats with the historically observed actual distributions (as shown in Figure 8). The figure clearly shows that the historical distribution records fall entirely within the currently predicted suitable habitats. This result not only significantly enhances the model's credibility but also provides a solid historical context to support the research findings, further validating the robustness and reliability of the model in predicting species distributions.

Climate factors are one of the key elements influencing the geographic distribution of *Termitomyces* species. Through a comprehensive analysis of the contribution rates of each climate factor and validation using the Jackknife test, we found that the cumulative contribution rate of precipitation factors is 72.8%, and the cumulative contribution rate of temperature factors is 24.9%. These two climate factors account for 97.7% of the total contribution from one terrain factor and 19 climate factors. This suggests that precipitation and temperature have a particularly significant effect on the geographic distribution of *Termitomyces* species.

Among them, the most significant climate factor is the precipitation during the warmest season (Bio18), with a contribution rate of 63.7% (Table 2). The response curve shows that when the precipitation during the warmest season is between 1187.8 and 1556.1 mm, the probability of occurrence of *Termitomyces* reaches its peak. Previous studies have also indicated that *Termitomyces* is able to fruit successfully in habitats with strong precipitation and suitable microclimate characteristics (Koné et al. 2011). Therefore, regions such as Guangxi, Guangdong, and Fujian, where precipitation exceeds 800 mm, in addition to Yunnan, Sichuan, Guizhou, and Chongqing, will also become suitable habitats for *Termitomyces* species. Additionally, the average temperature during the driest quarter (Bio09), temperature seasonality variance (Bio04), and the average temperature during the wettest season (Bio08) are also important factors influencing the distribution of *Termitomyces*. Related studies suggest that the optimal growth temperature for *Termitomyces* is 22°C–30°C (Wiriya et al. 2014), which means that temperature factors should not be neglected during artificial cultivation. In particular, temperature control should be strengthened in areas where the temperature falls below 22°C.

**FIGURE 9:**
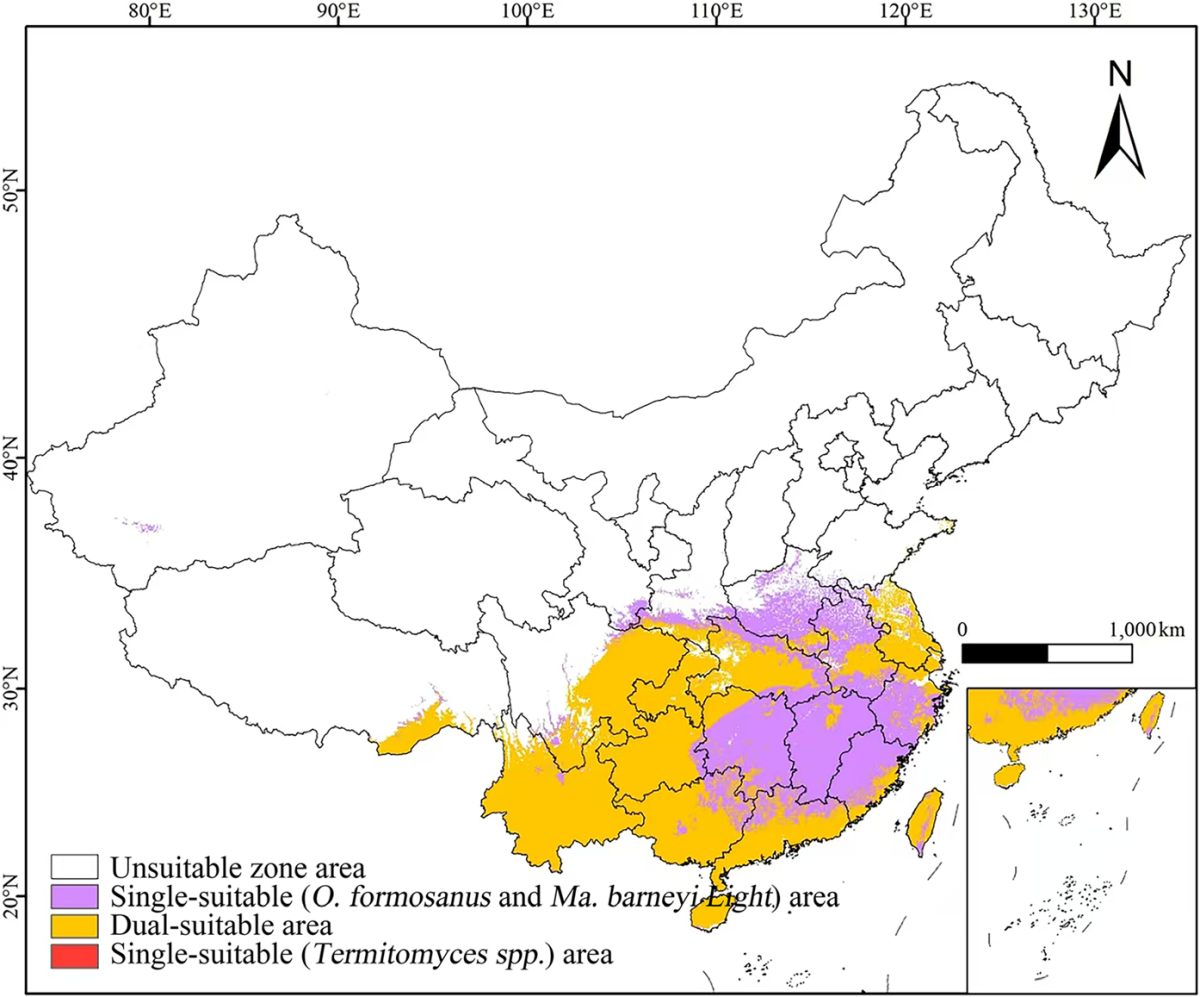
A comparison of the current potential suitable habitats of *Termitomyces* and two common species in Macrotermitinae— *O. formosanus* and Ma. barneyi Light. White, unsuitable zone area; purple, single‐suitable ( *O. formosanus* and Ma. barneyi Light) area; yellow, dual‐suitable area; red, single‐suitable (*Termitomyces* spp.) area.

In addition to the influence of climatic and topographic factors, Figure 9 shows a significant overlap (66.8%) between the current potential suitable habitats of *Termitomyces* spp. and those of two common species in the subfamily Macrotermitinae— *O. formosanus* and *Ma. barneyi Light*. (The total suitable habitat area for *O. formosanus* and *Ma. barneyi Light* is 228.93 × 10^4^ km^2^.) This quantitative result suggests that Macrotermitinae may play an important role in driving the expansion of Termitomyces spp.'s suitable habitats. Moreover, environmental factors such as soil pH, light intensity, and ventilation also influence the distribution of *Termitomyces* (Huiming et al. 2019). However, existing technologies are not yet fully developed, and it is currently not possible to integrate all influencing factors into a unified model to accurately simulate the potential distribution of the species. Nevertheless, this study provides important reference value for evaluating the potential suitable habitat of *Termitomyces* under climate change and for its artificial cultivation.

Under current climate conditions, the MaxEnt model's predicted results show that the total suitable habitat for *Termitomyces* is mainly concentrated in the southwestern, southern, central, and eastern regions of China. Specifically, the highly suitable habitat for *Termitomyces* is primarily located in the western Yunnan‐Guizhou Plateau and the Sichuan Basin in southwestern China. Taking the western Yunnan‐Guizhou Plateau as an example, this region has complex terrain with altitudes above 2000 m and is characterized by a subtropical monsoon climate, with an annual average temperature of 15.0°C–17.5°C and annual precipitation of 1100 mm. The warm and humid climate conditions are conducive to the growth of *Termitomyces* (Cheng et al. 2019; Jie et al. 2023; Zhuang et al. 2017). Therefore, the western Yunnan‐Guizhou Plateau could serve as an important ecological conservation area for *Termitomyces*, and its potential for nutrition and medicinal use could be further developed, leading to multi‐benefit exploitation.

Additionally, the Yunnan‐Guizhou Plateau and Sichuan Basin are also assessed as potential moderately suitable habitats for *Termitomyces*, where large‐scale artificial cultivation can be considered, along with research on improving its resistance to environmental stresses. Currently, provinces such as Yunnan and Guizhou have already begun related artificial cultivation technology research (Binbin et al. 2024; Tao et al. 2023; Wenguang et al. 2023). Although *Termitomyces* has only been found in limited natural distributions in provinces such as Guangxi, Guangdong, Hubei, and Jiangsu, large areas in these regions are potential low‐suitability habitats. Relevant authorities should focus on these areas for promoting and developing *Termitomyces*, encouraging local governments and research institutions to conduct further studies and demonstration planting to foster the development of local forest economy industries, whereas also promoting biodiversity conservation.

Under future climate warming scenarios, the areas of suitable habitats for *Termitomyces* significantly increase, primarily characterized by the westward shift of highly suitable areas and the eastward expansion of moderately and lowly suitable areas. There are two possible explanations for this trend: Firstly, climate warming leads to higher temperatures, which broaden the growth temperature range of *Termitomyces*, enabling it to successfully grow in regions that were previously too cold, thus pushing the highly suitable areas westward. This temperature change provides more favorable growing conditions for *Termitomyces*, particularly in some plateau areas where the rise in temperature creates opportunities for habitat expansion. Secondly, changes in precipitation and precipitation patterns also play a positive role in the distribution of *Termitomyces*. In the context of climate warming, some regions experience increased precipitation or changes in precipitation patterns, leading to higher humidity (Binbin et al. 2024; Li et al. 2012), which provides a more suitable moist environment for *Termitomyces*, facilitating the eastward expansion of moderately and lowly suitable areas.

However, despite the overall increase in suitable habitat areas, there are still some reductions in the moderately and lowly suitable areas under climate warming, indicating that the negative impacts of climate warming on the distribution of *Termitomyces* still persist. The main reason for this is the increasing frequency of extreme weather events triggered by future climate warming, including the effects on droughts and floods in China (Liu et al. 2010). These unstable climate factors pose a threat to the survival environment of *Termitomyces*. Although higher temperatures may provide more space for distribution, extreme weather conditions may render some habitats unsuitable, especially in areas dependent on stable water sources. Therefore, the impact of climate warming on the distribution of *Termitomyces* presents a complex dual effect, with both positive habitat expansion and potential environmental pressures.

Under different greenhouse gas emission scenarios, the trend in the changes to suitable habitats for *Termitomyces* also exhibits a dual effect. For instance, in the 2050s, under different emission scenarios, the area of highly suitable habitats in the SSP5‐8.5 scenario is larger than in the SSP2‐4.5 scenario, and the area in the SSP2‐4.5 scenario is larger than in the SSP1‐2.6 scenario. This indicates that an increase in greenhouse gas emissions will intensify climate warming, thereby expanding the range of suitable habitats. However, in the 2070s, the areas of low and moderately suitable habitats in the SSP5‐8.5 scenario are smaller than in the SSP2‐4.5 scenario, which means that future climate change not only promotes the expansion of suitable habitats for *Termitomyces*, but also brings about adverse effects.

Based on the above research findings, we recommend that future studies and predictions should comprehensively consider the combined effects of various climatic factors, including temperature, precipitation, and extreme climate events. Additionally, strategies should be developed to address potential extreme climate events. For example, optimizing the layout of cultivation areas and enhancing water resource management (Cubasch et al. 2001; Huo‐Po et al. 2013) may help mitigate the negative impacts of climate change on the habitats of *Termitomyces*, thereby maintaining the stability of its ecological suitability in the context of climate change.

This study provides valuable scientific evidence for the potential distribution of *Termitomyces* under the context of climate change. However, there are still certain limitations, and future research can further improve and expand in several areas. First, although this study predicts the potential distribution based on the MaxEnt model, ecological factors beyond climate variables, such as soil type, soil pH, vegetation cover, and microclimate conditions, have not been sufficiently considered (Gong and Guan 2020). Future work could integrate more ecological variables to develop more complex and comprehensive ecological models, thereby enhancing the accuracy and reliability of the predictions. Second, future research should combine field observation data to validate the accuracy of the model predictions. Long‐term monitoring of *Termitomyces* spp. distribution in different regions could provide more calibration data, thus improving the reliability and precision of the predictions. Lastly, although we have conducted a potential suitability assessment of *Termitomyces* under climate change, studies on its ecological adaptability are still limited. The adaptation mechanisms of *Termitomyces* to climate change, including tolerance to temperature, precipitation, and other factors, have not been fully explored. Future research could integrate experimental data to conduct physiological and ecological studies on *Termitomyces*, especially focusing on its resistance to stress, drought tolerance, and cold resistance, to provide a more scientific theoretical basis for its cultivation and conservation.

## 5 Conclusions

This study systematically predicted the potential distribution pattern of *Termitomyces* under the context of climate change using the MaxEnt model and ArcGIS software. By analyzing the relationship between the geographic distribution of *Termitomyces* and bioclimatic factors, the study revealed that temperature and precipitation are the key climatic factors affecting its distribution. The simulation results show that under current climate conditions, the total area of suitable habitat for *Termitomyces* reaches 152.95 × 10^4^ km^2^, mainly distributed in the southwest, south, central, and eastern regions of China. With future climate scenarios, the potential suitable habitats of *Termitomyces* are projected to gradually expand, even covering the northwest regions. The overall suitable habitat area significantly increases, with only a slight reduction in the area of moderately and low‐suitability regions. Additionally, the study found that the centroid of the suitable habitat is shifting toward the northwest. In conclusion, this research provides valuable scientific evidence for understanding the potential impact of climate change on the distribution of *Termitomyces*, optimizing its cultivation techniques, advancing ecological protection efforts, and formulating regional economic development strategies. It offers important guidance for research and practice in related fields.

## Author Contributions

**Zhihang Zhuo:** conceptualization (equal), formal analysis (equal), supervision (equal), writing – review and editing (equal). **Dan Yong:** formal analysis (equal), methodology (equal), writing – original draft (equal). **Danping Xu:** methodology (equal), writing – review and editing (equal). **Xinqi Deng:** data curation (equal). **Zhipeng He:** software (equal). **Biyu Liu:** software (equal). **Xuezhen Yang:** investigation (equal).

## Conflicts of Interest

The authors declare no conflicts of interest.

## Data Availability

The data supporting the results are available in a public repository at: https://doi.org/10.6084/m9.figshare.28094903.v1.
